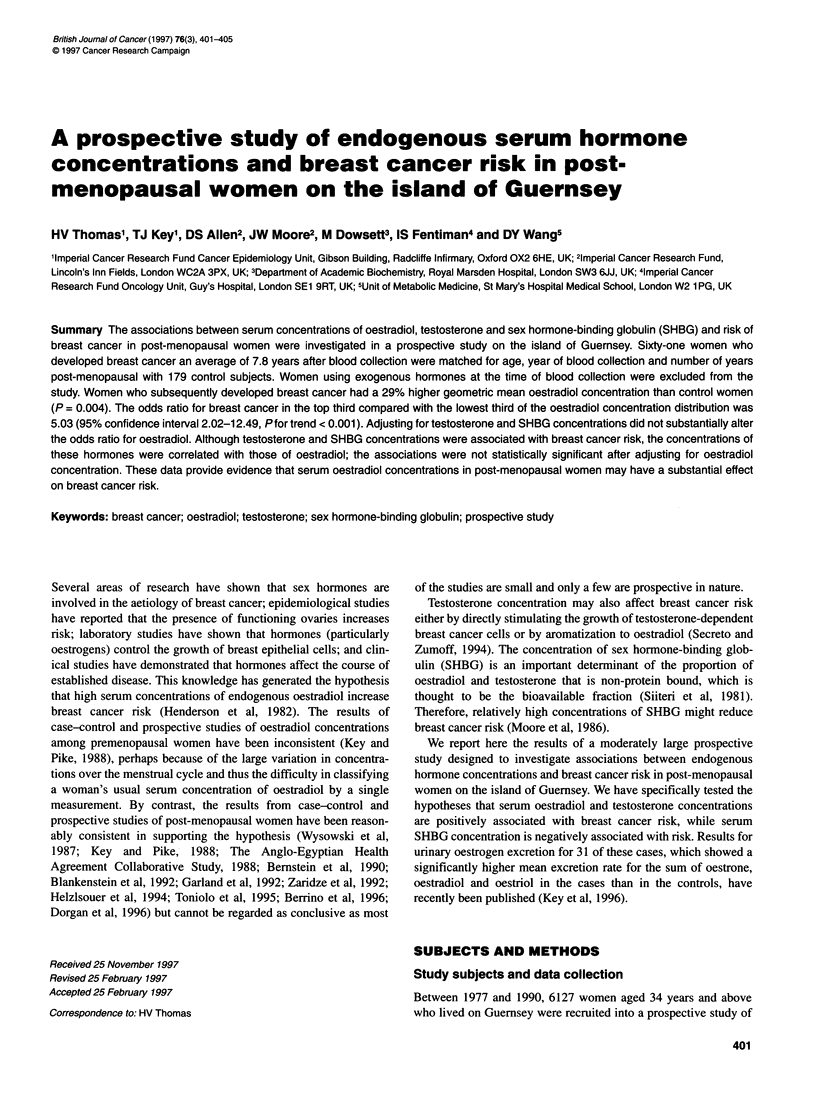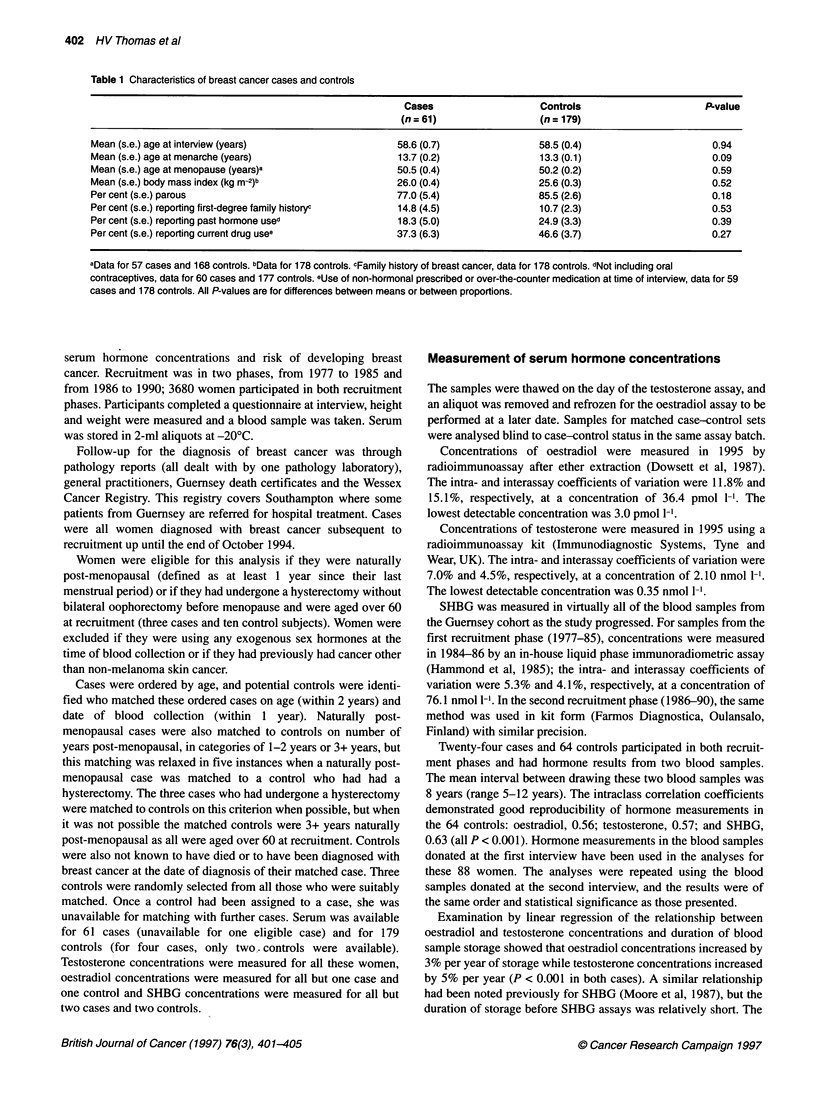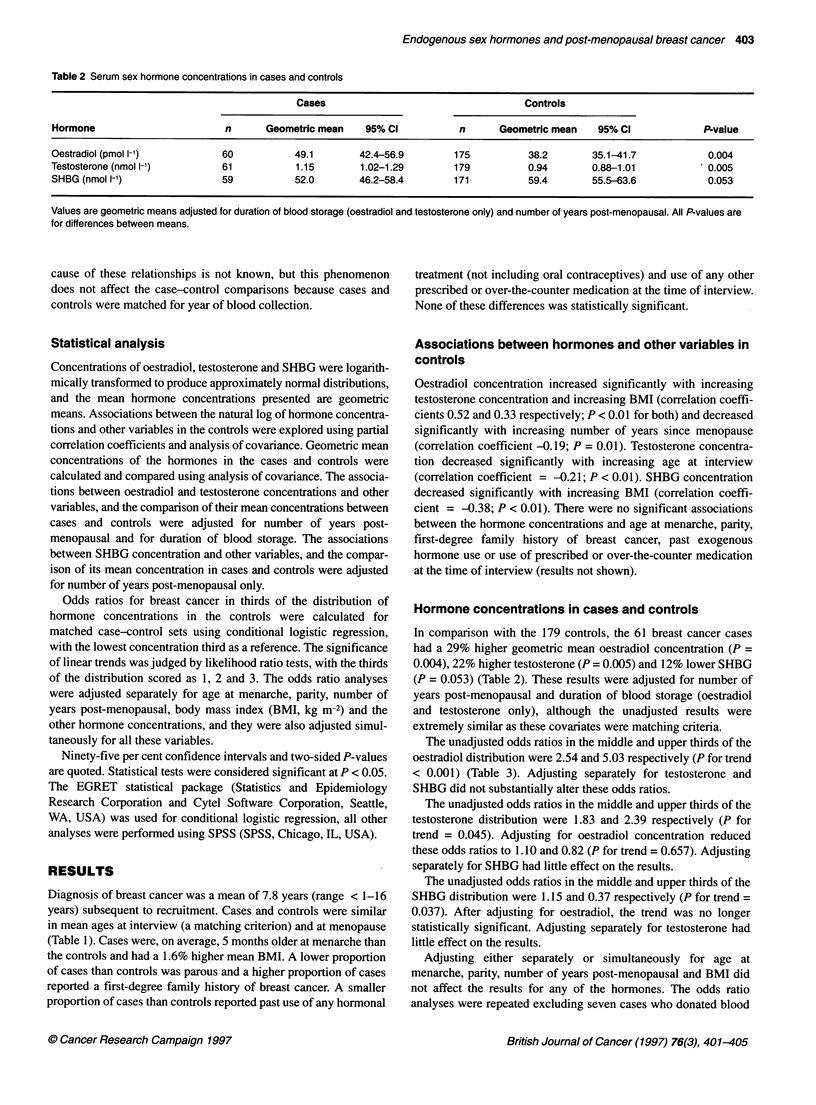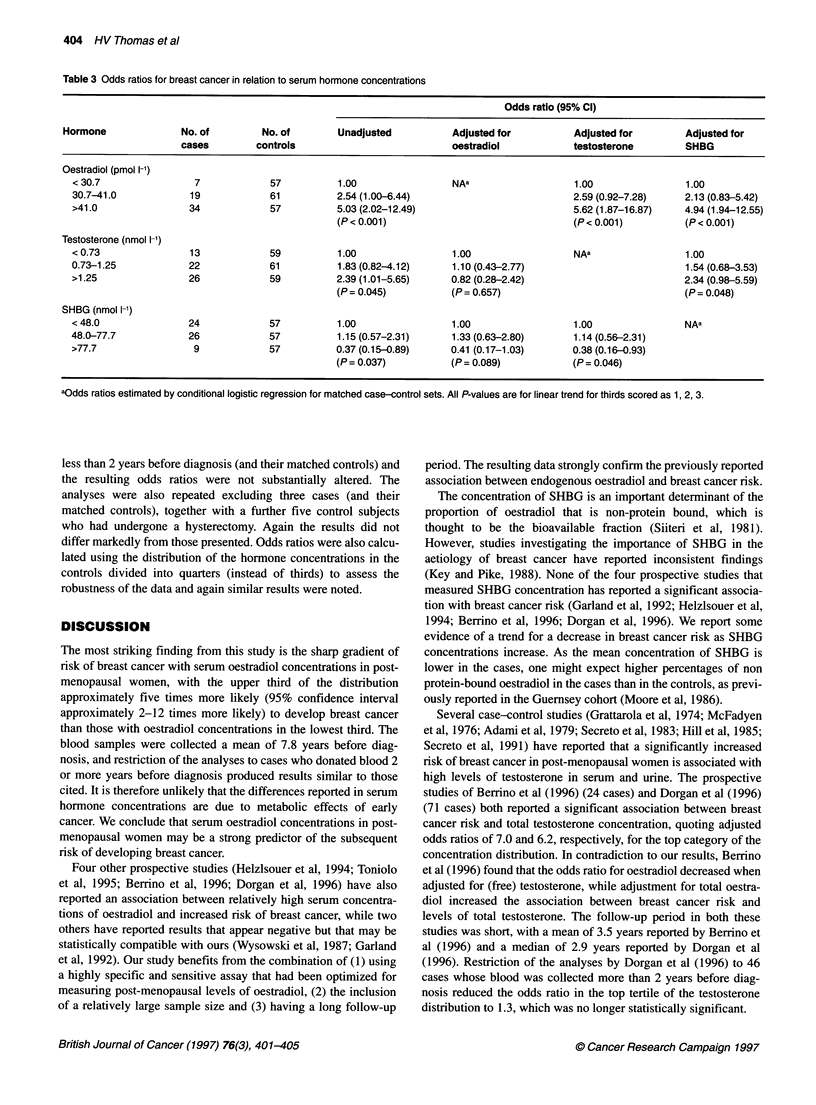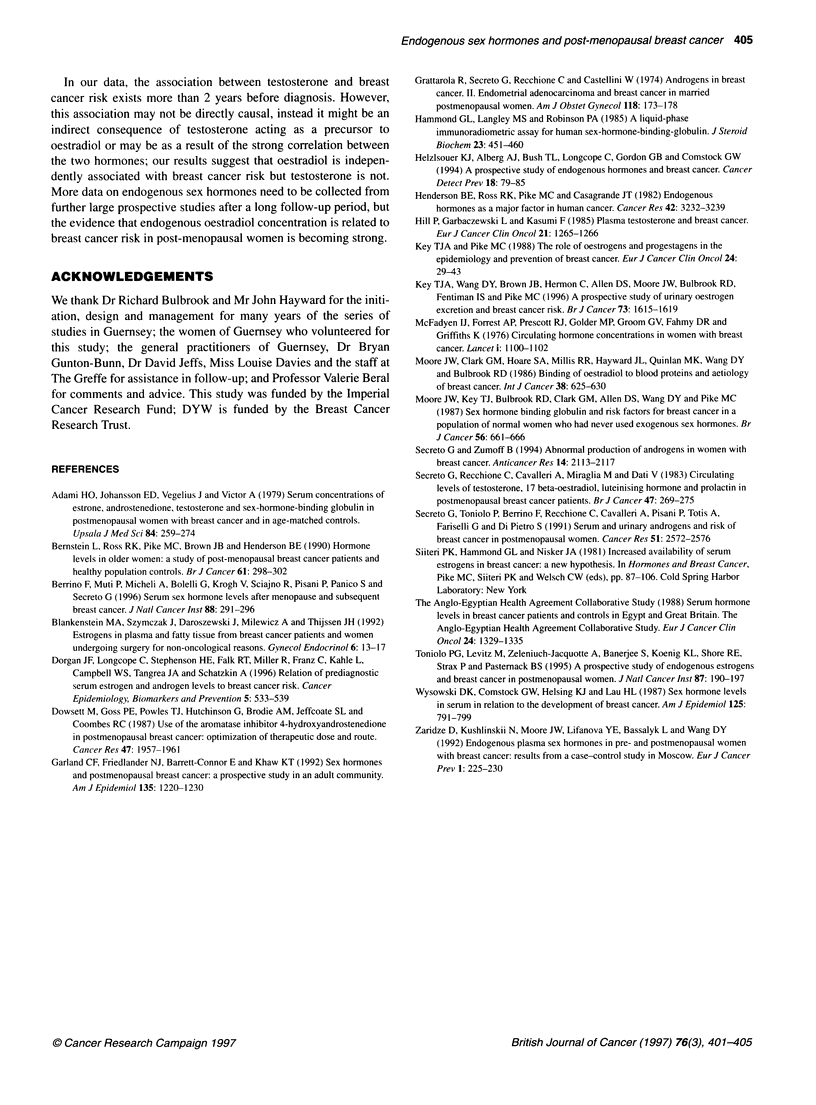# A prospective study of endogenous serum hormone concentrations and breast cancer risk in post-menopausal women on the island of Guernsey.

**DOI:** 10.1038/bjc.1997.398

**Published:** 1997

**Authors:** H. V. Thomas, T. J. Key, D. S. Allen, J. W. Moore, M. Dowsett, I. S. Fentiman, D. Y. Wang

**Affiliations:** Imperial Cancer Research Fund Cancer Epidemiology Unit, Radcliffe Infirmary, Oxford, UK.

## Abstract

The associations between serum concentrations of oestradiol, testosterone and sex hormone-binding globulin (SHBG) and risk of breast cancer in post-menopausal women were investigated in a prospective study on the island of Guernsey. Sixty-one women who developed breast cancer an average of 7.8 years after blood collection were matched for age, year of blood collection and number of years post-menopausal with 179 control subjects. Women using exogenous hormones at the time of blood collection were excluded from the study. Women who subsequently developed breast cancer had a 29% higher geometric mean oestradiol concentration than control women (P = 0.004). The odds ratio for breast cancer in the top third compared with the lowest third of the oestradiol concentration distribution was 5.03 (95% confidence interval 2.02-12.49, P for trend < 0.001). Adjusting for testosterone and SHBG concentrations did not substantially alter the odds ratio for oestradiol. Although testosterone and SHBG concentrations were associated with breast cancer risk, the concentrations of these hormones were correlated with those of oestradiol; the associations were not statistically significant after adjusting for oestradiol concentration. These data provide evidence that serum oestradiol concentrations in post-menopausal women may have a substantial effect on breast cancer risk.


*British Journal of Cancer* (1997) **76**(3), 401–405
© 1997 Cancer Research Campaign

### A prospective study of endogenous serum hormone concentrations and breast cancer risk in post-menopausal women on the island of Guernsey

**HV Thomas[1], TJ Key[1], DS Allen[2], JW Moore[2], M Dowsett[3], IS Fentiman[4] and DY Wang[5]**

[1]Imperial Cancer Research Fund Cancer Epidemiology Unit, Gibson Building, Radcliffe Infirmary, Oxford OX2 6HE, UK; [2]Imperial Cancer Research Fund, Lincoln's Inn Fields, London WC2A 3PX, UK; [3]Department of Academic Biochemistry, Royal Marsden Hospital, London SW3 6JJ, UK; [4]Imperial Cancer Research Fund Oncology Unit, Guy's Hospital, London SE1 9RT, UK; [5]Unit of Metabolic Medicine, St Mary's Hospital Medical School, London W2 1PG, UK

**Summary** The associations between serum concentrations of oestradiol, testosterone and sex hormone-binding globulin (SHBG) and risk of breast cancer in post-menopausal women were investigated in a prospective study on the island of Guernsey. Sixty-one women who developed breast cancer an average of 7.8 years after blood collection were matched for age, year of blood collection and number of years post-menopausal with 179 control subjects. Women using exogenous hormones at the time of blood collection were excluded from the study. Women who subsequently developed breast cancer had a 29% higher geometric mean oestradiol concentration than control women ($P = 0.004$). The odds ratio for breast cancer in the top third compared with the lowest third of the oestradiol concentration distribution was 5.03 (95% confidence interval 2.02–12.49, $P$ for trend $< 0.001$). Adjusting for testosterone and SHBG concentrations did not substantially alter the odds ratio for oestradiol. Although testosterone and SHBG concentrations were associated with breast cancer risk, the concentrations of these hormones were correlated with those of oestradiol; the associations were not statistically significant after adjusting for oestradiol concentration. These data provide evidence that serum oestradiol concentrations in post-menopausal women may have a substantial effect on breast cancer risk.

**Keywords:** breast cancer; oestradiol; testosterone; sex hormone-binding globulin; prospective study

Several areas of research have shown that sex hormones are involved in the aetiology of breast cancer; epidemiological studies have reported that the presence of functioning ovaries increases risk; laboratory studies have shown that hormones (particularly oestrogens) control the growth of breast epithelial cells; and clinical studies have demonstrated that hormones affect the course of established disease. This knowledge has generated the hypothesis that high serum concentrations of endogenous oestradiol increase breast cancer risk (Henderson et al, 1982). The results of case–control and prospective studies of oestradiol concentrations among premenopausal women have been inconsistent (Key and Pike, 1988), perhaps because of the large variation in concentrations over the menstrual cycle and thus the difficulty in classifying a woman's usual serum concentration of oestradiol by a single measurement. By contrast, the results from case–control and prospective studies of post-menopausal women have been reasonably consistent in supporting the hypothesis (Wysowski et al, 1987; Key and Pike, 1988; The Anglo-Egyptian Health Agreement Collaborative Study, 1988; Bernstein et al, 1990; Blankenstein et al, 1992; Garland et al, 1992; Zaridze et al, 1992; Helzlsouer et al, 1994; Toniolo et al, 1995; Berrino et al, 1996; Dorgan et al, 1996) but cannot be regarded as conclusive as most of the studies are small and only a few are prospective in nature.

Testosterone concentration may also affect breast cancer risk either by directly stimulating the growth of testosterone-dependent breast cancer cells or by aromatization to oestradiol (Secreto and Zumoff, 1994). The concentration of sex hormone-binding globulin (SHBG) is an important determinant of the proportion of oestradiol and testosterone that is non-protein bound, which is thought to be the bioavailable fraction (Siiteri et al, 1981). Therefore, relatively high concentrations of SHBG might reduce breast cancer risk (Moore et al, 1986).

We report here the results of a moderately large prospective study designed to investigate associations between endogenous hormone concentrations and breast cancer risk in post-menopausal women on the island of Guernsey. We have specifically tested the hypotheses that serum oestradiol and testosterone concentrations are positively associated with breast cancer risk, while serum SHBG concentration is negatively associated with risk. Results for urinary oestrogen excretion for 31 of these cases, which showed a significantly higher mean excretion rate for the sum of oestrone, oestradiol and oestriol in the cases than in the controls, have recently been published (Key et al, 1996).

*Received 25 November 1997*
*Revised 25 February 1997*
*Accepted 25 February 1997*

*Correspondence to:* HV Thomas

### SUBJECTS AND METHODS

### Study subjects and data collection

Between 1977 and 1990, 6127 women aged 34 years and above who lived on Guernsey were recruited into a prospective study of

**Table 1** Characteristics of breast cancer cases and controls

| | Cases ($n$ = 61) | Controls ($n$ = 179) | $P$-value |
|---|---|---|---|
| Mean (s.e.) age at interview (years) | 58.6 (0.7) | 58.5 (0.4) | 0.94 |
| Mean (s.e.) age at menarche (years) | 13.7 (0.2) | 13.3 (0.1) | 0.09 |
| Mean (s.e.) age at menopause (years)[a] | 50.5 (0.4) | 50.2 (0.2) | 0.59 |
| Mean (s.e.) body mass index (kg $m^{-2}$)[b] | 26.0 (0.4) | 25.6 (0.3) | 0.52 |
| Per cent (s.e.) parous | 77.0 (5.4) | 85.5 (2.6) | 0.18 |
| Per cent (s.e.) reporting first-degree family history[c] | 14.8 (4.5) | 10.7 (2.3) | 0.53 |
| Per cent (s.e.) reporting past hormone use[d] | 18.3 (5.0) | 24.9 (3.3) | 0.39 |
| Per cent (s.e.) reporting current drug use[e] | 37.3 (6.3) | 46.6 (3.7) | 0.27 |

[a]Data for 57 cases and 168 controls. [b]Data for 178 controls. [c]Family history of breast cancer, data for 178 controls. [d]Not including oral contraceptives, data for 60 cases and 177 controls. [e]Use of non-hormonal prescribed or over-the-counter medication at time of interview, data for 59 cases and 178 controls. All $P$-values are for differences between means or between proportions.

serum hormone concentrations and risk of developing breast cancer. Recruitment was in two phases, from 1977 to 1985 and from 1986 to 1990; 3680 women participated in both recruitment phases. Participants completed a questionnaire at interview, height and weight were measured and a blood sample was taken. Serum was stored in 2-ml aliquots at –20°C.

Follow-up for the diagnosis of breast cancer was through pathology reports (all dealt with by one pathology laboratory), general practitioners, Guernsey death certificates and the Wessex Cancer Registry. This registry covers Southampton where some patients from Guernsey are referred for hospital treatment. Cases were all women diagnosed with breast cancer subsequent to recruitment up until the end of October 1994.

Women were eligible for this analysis if they were naturally post-menopausal (defined as at least 1 year since their last menstrual period) or if they had undergone a hysterectomy without bilateral oophorectomy before menopause and were aged over 60 at recruitment (three cases and ten control subjects). Women were excluded if they were using any exogenous sex hormones at the time of blood collection or if they had previously had cancer other than non-melanoma skin cancer.

Cases were ordered by age, and potential controls were identified who matched these ordered cases on age (within 2 years) and date of blood collection (within 1 year). Naturally post-menopausal cases were also matched to controls on number of years post-menopausal, in categories of 1–2 years or 3+ years, but this matching was relaxed in five instances when a naturally post-menopausal case was matched to a control who had had a hysterectomy. The three cases who had undergone a hysterectomy were matched to controls on this criterion when possible, but when it was not possible the matched controls were 3+ years naturally post-menopausal as all were aged over 60 at recruitment. Controls were also not known to have died or to have been diagnosed with breast cancer at the date of diagnosis of their matched case. Three controls were randomly selected from all those who were suitably matched. Once a control had been assigned to a case, she was unavailable for matching with further cases. Serum was available for 61 cases (unavailable for one eligible case) and for 179 controls (for four cases, only two controls were available). Testosterone concentrations were measured for all these women, oestradiol concentrations were measured for all but one case and one control and SHBG concentrations were measured for all but two cases and two controls.

### Measurement of serum hormone concentrations

The samples were thawed on the day of the testosterone assay, and an aliquot was removed and refrozen for the oestradiol assay to be performed at a later date. Samples for matched case–control sets were analysed blind to case–control status in the same assay batch.

Concentrations of oestradiol were measured in 1995 by radioimmunoassay after ether extraction (Dowsett et al, 1987). The intra- and interassay coefficients of variation were 11.8% and 15.1%, respectively, at a concentration of 36.4 pmol $l^{-1}$. The lowest detectable concentration was 3.0 pmol $l^{-1}$.

Concentrations of testosterone were measured in 1995 using a radioimmunoassay kit (Immunodiagnostic Systems, Tyne and Wear, UK). The intra- and interassay coefficients of variation were 7.0% and 4.5%, respectively, at a concentration of 2.10 nmol $l^{-1}$. The lowest detectable concentration was 0.35 nmol $l^{-1}$.

SHBG was measured in virtually all of the blood samples from the Guernsey cohort as the study progressed. For samples from the first recruitment phase (1977–85), concentrations were measured in 1984–86 by an in-house liquid phase immunoradiometric assay (Hammond et al, 1985); the intra- and interassay coefficients of variation were 5.3% and 4.1%, respectively, at a concentration of 76.1 nmol $l^{-1}$. In the second recruitment phase (1986–90), the same method was used in kit form (Farmos Diagnostica, Oulansalo, Finland) with similar precision.

Twenty-four cases and 64 controls participated in both recruitment phases and had hormone results from two blood samples. The mean interval between drawing these two blood samples was 8 years (range 5–12 years). The intraclass correlation coefficients demonstrated good reproducibility of hormone measurements in the 64 controls: oestradiol, 0.56; testosterone, 0.57; and SHBG, 0.63 (all $P < 0.001$). Hormone measurements in the blood samples donated at the first interview have been used in the analyses for these 88 women. The analyses were repeated using the blood samples donated at the second interview, and the results were of the same order and statistical significance as those presented.

Examination by linear regression of the relationship between oestradiol and testosterone concentrations and duration of blood sample storage showed that oestradiol concentrations increased by 3% per year of storage while testosterone concentrations increased by 5% per year ($P < 0.001$ in both cases). A similar relationship had been noted previously for SHBG (Moore et al, 1987), but the duration of storage before SHBG assays was relatively short. The

© Cancer Research Campaign 1997

**Table 2** Serum sex hormone concentrations in cases and controls

| | Cases | | | Controls | | | |
|---|---|---|---|---|---|---|---|
| **Hormone** | *n* | **Geometric mean** | **95% CI** | *n* | **Geometric mean** | **95% CI** | ***P*-value** |
| Oestradiol (pmol $l^{-1}$) | 60 | 49.1 | 42.4–56.9 | 175 | 38.2 | 35.1–41.7 | 0.004 |
| Testosterone (nmol $l^{-1}$) | 61 | 1.15 | 1.02–1.29 | 179 | 0.94 | 0.88–1.01 | 0.005 |
| SHBG (nmol $l^{-1}$) | 59 | 52.0 | 46.2–58.4 | 171 | 59.4 | 55.5–63.6 | 0.053 |

Values are geometric means adjusted for duration of blood storage (oestradiol and testosterone only) and number of years post-menopausal. All *P*-values are for differences between means.

cause of these relationships is not known, but this phenomenon does not affect the case–control comparisons because cases and controls were matched for year of blood collection.

### Statistical analysis

Concentrations of oestradiol, testosterone and SHBG were logarithmically transformed to produce approximately normal distributions, and the mean hormone concentrations presented are geometric means. Associations between the natural log of hormone concentrations and other variables in the controls were explored using partial correlation coefficients and analysis of covariance. Geometric mean concentrations of the hormones in the cases and controls were calculated and compared using analysis of covariance. The associations between oestradiol and testosterone concentrations and other variables, and the comparison of their mean concentrations between cases and controls were adjusted for number of years post-menopausal and for duration of blood storage. The associations between SHBG concentration and other variables, and the comparison of its mean concentration in cases and controls were adjusted for number of years post-menopausal only.

Odds ratios for breast cancer in thirds of the distribution of hormone concentrations in the controls were calculated for matched case–control sets using conditional logistic regression, with the lowest concentration third as a reference. The significance of linear trends was judged by likelihood ratio tests, with the thirds of the distribution scored as 1, 2 and 3. The odds ratio analyses were adjusted separately for age at menarche, parity, number of years post-menopausal, body mass index (BMI, kg $m^{-2}$) and the other hormone concentrations, and they were also adjusted simultaneously for all these variables.

Ninety-five per cent confidence intervals and two-sided *P*-values are quoted. Statistical tests were considered significant at $P < 0.05$. The EGRET statistical package (Statistics and Epidemiology Research Corporation and Cytel Software Corporation, Seattle, WA, USA) was used for conditional logistic regression, all other analyses were performed using SPSS (SPSS, Chicago, IL, USA).

### RESULTS

Diagnosis of breast cancer was a mean of 7.8 years (range < 1–16 years) subsequent to recruitment. Cases and controls were similar in mean ages at interview (a matching criterion) and at menopause (Table 1). Cases were, on average, 5 months older at menarche than the controls and had a 1.6% higher mean BMI. A lower proportion of cases than controls was parous and a higher proportion of cases reported a first-degree family history of breast cancer. A smaller proportion of cases than controls reported past use of any hormonal treatment (not including oral contraceptives) and use of any other prescribed or over-the-counter medication at the time of interview. None of these differences was statistically significant.

### Associations between hormones and other variables in controls

Oestradiol concentration increased significantly with increasing testosterone concentration and increasing BMI (correlation coefficients 0.52 and 0.33 respectively; $P < 0.01$ for both) and decreased significantly with increasing number of years since menopause (correlation coefficient –0.19; $P = 0.01$). Testosterone concentration decreased significantly with increasing age at interview (correlation coefficient = –0.21; $P < 0.01$). SHBG concentration decreased significantly with increasing BMI (correlation coefficient = –0.38; $P < 0.01$). There were no significant associations between the hormone concentrations and age at menarche, parity, first-degree family history of breast cancer, past exogenous hormone use or use of prescribed or over-the-counter medication at the time of interview (results not shown).

### Hormone concentrations in cases and controls

In comparison with the 179 controls, the 61 breast cancer cases had a 29% higher geometric mean oestradiol concentration ($P = 0.004$), 22% higher testosterone ($P = 0.005$) and 12% lower SHBG ($P = 0.053$) (Table 2). These results were adjusted for number of years post-menopausal and duration of blood storage (oestradiol and testosterone only), although the unadjusted results were extremely similar as these covariates were matching criteria.

The unadjusted odds ratios in the middle and upper thirds of the oestradiol distribution were 2.54 and 5.03 respectively (*P* for trend < 0.001) (Table 3). Adjusting separately for testosterone and SHBG did not substantially alter these odds ratios.

The unadjusted odds ratios in the middle and upper thirds of the testosterone distribution were 1.83 and 2.39 respectively (*P* for trend = 0.045). Adjusting for oestradiol concentration reduced these odds ratios to 1.10 and 0.82 (*P* for trend = 0.657). Adjusting separately for SHBG had little effect on the results.

The unadjusted odds ratios in the middle and upper thirds of the SHBG distribution were 1.15 and 0.37 respectively (*P* for trend = 0.037). After adjusting for oestradiol, the trend was no longer statistically significant. Adjusting separately for testosterone had little effect on the results.

Adjusting either separately or simultaneously for age at menarche, parity, number of years post-menopausal and BMI did not affect the results for any of the hormones. The odds ratio analyses were repeated excluding seven cases who donated blood

© Cancer Research Campaign 1997

**Table 3** Odds ratios for breast cancer in relation to serum hormone concentrations

| Hormone | No. of cases | No. of controls | Odds ratio (95% CI) Unadjusted | Adjusted for oestradiol | Adjusted for testosterone | Adjusted for SHBG |
|---|---|---|---|---|---|---|
| Oestradiol (pmol $l^{-1}$) | | | | | | |
| < 30.7 | 7 | 57 | 1.00 | NA[a] | 1.00 | 1.00 |
| 30.7–41.0 | 19 | 61 | 2.54 (1.00–6.44) | | 2.59 (0.92–7.28) | 2.13 (0.83–5.42) |
| >41.0 | 34 | 57 | 5.03 (2.02–12.49) ($P$ < 0.001) | | 5.62 (1.87–16.87) ($P$ < 0.001) | 4.94 (1.94–12.55) ($P$ < 0.001) |
| Testosterone (nmol $l^{-1}$) | | | | | | |
| < 0.73 | 13 | 59 | 1.00 | 1.00 | NA[a] | 1.00 |
| 0.73–1.25 | 22 | 61 | 1.83 (0.82–4.12) | 1.10 (0.43–2.77) | | 1.54 (0.68–3.53) |
| >1.25 | 26 | 59 | 2.39 (1.01–5.65) ($P$ = 0.045) | 0.82 (0.28–2.42) ($P$ = 0.657) | | 2.34 (0.98–5.59) ($P$ = 0.048) |
| SHBG (nmol $l^{-1}$) | | | | | | |
| < 48.0 | 24 | 57 | 1.00 | 1.00 | 1.00 | NA[a] |
| 48.0–77.7 | 26 | 57 | 1.15 (0.57–2.31) | 1.33 (0.63–2.80) | 1.14 (0.56–2.31) | |
| >77.7 | 9 | 57 | 0.37 (0.15–0.89) ($P$ = 0.037) | 0.41 (0.17–1.03) ($P$ = 0.089) | 0.38 (0.16–0.93) ($P$ = 0.046) | |

[a]Odds ratios estimated by conditional logistic regression for matched case–control sets. All $P$-values are for linear trend for thirds scored as 1, 2, 3.

less than 2 years before diagnosis (and their matched controls) and the resulting odds ratios were not substantially altered. The analyses were also repeated excluding three cases (and their matched controls), together with a further five control subjects who had undergone a hysterectomy. Again the results did not differ markedly from those presented. Odds ratios were also calculated using the distribution of the hormone concentrations in the controls divided into quarters (instead of thirds) to assess the robustness of the data and again similar results were noted.

### DISCUSSION

The most striking finding from this study is the sharp gradient of risk of breast cancer with serum oestradiol concentrations in post-menopausal women, with the upper third of the distribution approximately five times more likely (95% confidence interval approximately 2–12 times more likely) to develop breast cancer than those with oestradiol concentrations in the lowest third. The blood samples were collected a mean of 7.8 years before diagnosis, and restriction of the analyses to cases who donated blood 2 or more years before diagnosis produced results similar to those cited. It is therefore unlikely that the differences reported in serum hormone concentrations are due to metabolic effects of early cancer. We conclude that serum oestradiol concentrations in post-menopausal women may be a strong predictor of the subsequent risk of developing breast cancer.

Four other prospective studies (Helzlsouer et al, 1994; Toniolo et al, 1995; Berrino et al, 1996; Dorgan et al, 1996) have also reported an association between relatively high serum concentrations of oestradiol and increased risk of breast cancer, while two others have reported results that appear negative but that may be statistically compatible with ours (Wysowski et al, 1987; Garland et al, 1992). Our study benefits from the combination of (1) using a highly specific and sensitive assay that had been optimized for measuring post-menopausal levels of oestradiol, (2) the inclusion of a relatively large sample size and (3) having a long follow-up period. The resulting data strongly confirm the previously reported association between endogenous oestradiol and breast cancer risk.

The concentration of SHBG is an important determinant of the proportion of oestradiol that is non-protein bound, which is thought to be the bioavailable fraction (Siiteri et al, 1981). However, studies investigating the importance of SHBG in the aetiology of breast cancer have reported inconsistent findings (Key and Pike, 1988). None of the four prospective studies that measured SHBG concentration has reported a significant association with breast cancer risk (Garland et al, 1992; Helzlsouer et al, 1994; Berrino et al, 1996; Dorgan et al, 1996). We report some evidence of a trend for a decrease in breast cancer risk as SHBG concentrations increase. As the mean concentration of SHBG is lower in the cases, one might expect higher percentages of non protein-bound oestradiol in the cases than in the controls, as previously reported in the Guernsey cohort (Moore et al, 1986).

Several case–control studies (Grattarola et al, 1974; McFadyen et al, 1976; Adami et al, 1979; Secreto et al, 1983; Hill et al, 1985; Secreto et al, 1991) have reported that a significantly increased risk of breast cancer in post-menopausal women is associated with high levels of testosterone in serum and urine. The prospective studies of Berrino et al (1996) (24 cases) and Dorgan et al (1996) (71 cases) both reported a significant association between breast cancer risk and total testosterone concentration, quoting adjusted odds ratios of 7.0 and 6.2, respectively, for the top category of the concentration distribution. In contradiction to our results, Berrino et al (1996) found that the odds ratio for oestradiol decreased when adjusted for (free) testosterone, while adjustment for total oestradiol increased the association between breast cancer risk and levels of total testosterone. The follow-up period in both these studies was short, with a mean of 3.5 years reported by Berrino et al (1996) and a median of 2.9 years reported by Dorgan et al (1996). Restriction of the analyses by Dorgan et al (1996) to 46 cases whose blood was collected more than 2 years before diagnosis reduced the odds ratio in the top tertile of the testosterone distribution to 1.3, which was no longer statistically significant.

 © Cancer Research Campaign 1997

In our data, the association between testosterone and breast cancer risk exists more than 2 years before diagnosis. However, this association may not be directly causal, instead it might be an indirect consequence of testosterone acting as a precursor to oestradiol or may be as a result of the strong correlation between the two hormones; our results suggest that oestradiol is independently associated with breast cancer risk but testosterone is not. More data on endogenous sex hormones need to be collected from further large prospective studies after a long follow-up period, but the evidence that endogenous oestradiol concentration is related to breast cancer risk in post-menopausal women is becoming strong.

### ACKNOWLEDGEMENTS

We thank Dr Richard Bulbrook and Mr John Hayward for the initiation, design and management for many years of the series of studies in Guernsey; the women of Guernsey who volunteered for this study; the general practitioners of Guernsey, Dr Bryan Gunton-Bunn, Dr David Jeffs, Miss Louise Davies and the staff at The Greffe for assistance in follow-up; and Professor Valerie Beral for comments and advice. This study was funded by the Imperial Cancer Research Fund; DYW is funded by the Breast Cancer Research Trust.

### REFERENCES

Adami HO, Johansson ED, Vegelius J and Victor A (1979) Serum concentrations of estrone, androstenedione, testosterone and sex-hormone-binding globulin in postmenopausal women with breast cancer and in age-matched controls. *Upsala J Med Sci* **84**: 259–274

Bernstein L, Ross RK, Pike MC, Brown JB and Henderson BE (1990) Hormone levels in older women: a study of post-menopausal breast cancer patients and healthy population controls. *Br J Cancer* **61**: 298–302

Berrino F, Muti P, Micheli A, Bolelli G, Krogh V, Sciajno R, Pisani P, Panico S and Secreto G (1996) Serum sex hormone levels after menopause and subsequent breast cancer. *J Natl Cancer Inst* **88**: 291–296

Blankenstein MA, Szymczak J, Daroszewski J, Milewicz A and Thijssen JH (1992) Estrogens in plasma and fatty tissue from breast cancer patients and women undergoing surgery for non-oncological reasons. *Gynecol Endocrinol* **6**: 13–17

Dorgan JF, Longcope C, Stephenson HE, Falk RT, Miller R, Franz C, Kahle L, Campbell WS, Tangrea JA and Schatzkin A (1996) Relation of prediagnostic serum estrogen and androgen levels to breast cancer risk. *Cancer Epidemiology, Biomarkers and Prevention* **5**: 533–539

Dowsett M, Goss PE, Powles TJ, Hutchinson G, Brodie AM, Jeffcoate SL and Coombes RC (1987) Use of the aromatase inhibitor 4-hydroxyandrostenedione in postmenopausal breast cancer: optimization of therapeutic dose and route. *Cancer Res* **47**: 1957–1961

Garland CF, Friedlander NJ, Barrett-Connor E and Khaw KT (1992) Sex hormones and postmenopausal breast cancer: a prospective study in an adult community. *Am J Epidemiol* **135**: 1220–1230

Grattarola R, Secreto G, Recchione C and Castellini W (1974) Androgens in breast cancer. II. Endometrial adenocarcinoma and breast cancer in married postmenopausal women. *Am J Obstet Gynecol* **118**: 173–178

Hammond GL, Langley MS and Robinson PA (1985) A liquid-phase immunoradiometric assay for human sex-hormone-binding-globulin. *J Steroid Biochem* **23**: 451–460

Helzlsouer KJ, Alberg AJ, Bush TL, Longcope C, Gordon GB and Comstock GW (1994) A prospective study of endogenous hormones and breast cancer. *Cancer Detect Prev* **18**: 79–85

Henderson BE, Ross RK, Pike MC and Casagrande JT (1982) Endogenous hormones as a major factor in human cancer. *Cancer Res* **42**: 3232–3239

Hill P, Garbaczewski L and Kasumi F (1985) Plasma testosterone and breast cancer. *Eur J Cancer Clin Oncol* **21**: 1265–1266

Key TJA and Pike MC (1988) The role of oestrogens and progestagens in the epidemiology and prevention of breast cancer. *Eur J Cancer Clin Oncol* **24**: 29–43

Key TJA, Wang DY, Brown JB, Hermon C, Allen DS, Moore JW, Bulbrook RD, Fentiman IS and Pike MC (1996) A prospective study of urinary oestrogen excretion and breast cancer risk. *Br J Cancer* **73**: 1615–1619

McFadyen IJ, Forrest AP, Prescott RJ, Golder MP, Groom GV, Fahmy DR and Griffiths K (1976) Circulating hormone concentrations in women with breast cancer. *Lancet* **i**: 1100–1102

Moore JW, Clark GM, Hoare SA, Millis RR, Hayward JL, Quinlan MK, Wang DY and Bulbrook RD (1986) Binding of oestradiol to blood proteins and aetiology of breast cancer. *Int J Cancer* **38**: 625–630

Moore JW, Key TJ, Bulbrook RD, Clark GM, Allen DS, Wang DY and Pike MC (1987) Sex hormone binding globulin and risk factors for breast cancer in a population of normal women who had never used exogenous sex hormones. *Br J Cancer* **56**: 661–666

Secreto G and Zumoff B (1994) Abnormal production of androgens in women with breast cancer. *Anticancer Res* **14**: 2113–2117

Secreto G, Recchione C, Cavalleri A, Miraglia M and Dati V (1983) Circulating levels of testosterone, 17 beta-oestradiol, luteinising hormone and prolactin in postmenopausal breast cancer patients. *Br J Cancer* **47**: 269–275

Secreto G, Toniolo P, Berrino F, Recchione C, Cavalleri A, Pisani P, Totis A, Fariselli G and Di Pietro S (1991) Serum and urinary androgens and risk of breast cancer in postmenopausal women. *Cancer Res* **51**: 2572–2576

Siiteri PK, Hammond GL and Nisker JA (1981) Increased availability of serum estrogens in breast cancer: a new hypothesis. In *Hormones and Breast Cancer*, Pike MC, Siiteri PK and Welsch CW (eds), pp. 87–106. Cold Spring Harbor Laboratory: New York

The Anglo-Egyptian Health Agreement Collaborative Study (1988) Serum hormone levels in breast cancer patients and controls in Egypt and Great Britain. The Anglo-Egyptian Health Agreement Collaborative Study. *Eur J Cancer Clin Oncol* **24**: 1329–1335

Toniolo PG, Levitz M, Zeleniuch-Jacquotte A, Banerjee S, Koenig KL, Shore RE, Strax P and Pasternack BS (1995) A prospective study of endogenous estrogens and breast cancer in postmenopausal women. *J Natl Cancer Inst* **87**: 190–197

Wysowski DK, Comstock GW, Helsing KJ and Lau HL (1987) Sex hormone levels in serum in relation to the development of breast cancer. *Am J Epidemiol* **125**: 791–799

Zaridze D, Kushlinskii N, Moore JW, Lifanova YE, Bassalyk L and Wang DY (1992) Endogenous plasma sex hormones in pre- and postmenopausal women with breast cancer: results from a case–control study in Moscow. *Eur J Cancer Prev* **1**: 225–230

© Cancer Research Campaign 1997